# Analysis of influencing factors and scales of agglomerate fog on expressways

**DOI:** 10.1371/journal.pone.0324010

**Published:** 2025-06-12

**Authors:** Hongna Dai, Jingwen Wang, Haixia Feng, Vladimir Zyryanov, Zhongke Feng, Jipeng Cui

**Affiliations:** 1 Don College of Shandong Jiaotong University, Jinan, Shandong, China; 2 School of Transportation and Logistics Engineering, Shandong Jiaotong University, Jinan, Shandong, China; 3 Don State Technical University, Rostov-on-Don, Rostov Oblast, Russia; 4 Beijing Key Laboratory of Precision Forestry, Beijing Forestry University, Beijing, China; 5 Shandong Provincial Communications Planning and Design Institute Group Co., Ltd., Jinan, Shandong, China; Tongji University, CHINA

## Abstract

To enhance the monitoring accuracy of agglomerate fog on expressways, this paper takes the frequently occurring agglomerate fog data on Shandong’s expressways as an example. Based on the analysis of the spatiotemporal distribution characteristics of agglomerate fog, from the spatial perspective, it employs Geographic Weighted Regression (GWR) and Multi-scale Geographic Weighted Regression (MGWR) models to analyze the influence and scale of factors including Digital Elevation Model (DEM), DEM difference, water system density, Normalized Difference Vegetation Index (NDVI), Land Surface Temperature (LST) difference, and precipitation on agglomerate fog. The main research conclusions are as follows: agglomerate fog frequently occurred in the early morning during autumn and winter when the temperature difference is large. Three concentration centers of agglomerate fog-prone road segments were identified along Shandong’s expressways, located near Jiaozhou Bay, within intermountain basins of the central region, and across the northern plain of Mount Tai (where the Yellow River traverses the concentration center). The impacts of various influencing factors on agglomerate fog are ranked as follows: DEM > DEM difference > LST difference > water system density > NDVI > precipitation, among which DEM difference and LST difference mainly promote fog formation, whereas other factors generally exhibit inhibitory effect. The influence range (adaptive scale) of precipitation is the largest, at 673 meters, followed by the water system with an influence range of 599 meters, and NDVI shows the smallest influence range at only 44 meters. It holds significant importance for reducing the accident rate on expressways.

## Introduction

By the end of 2024, the total mileage of expressways in China had reached 185,000 kilometers, ranking first in the world in terms of road network scale. According to the data from the ’China Statistical Yearbook 2024’, there were 255,000 road traffic accidents in China in 2023, resulting in over 60,000 deaths. Among these, expressway traffic accidents account for a significant proportion, with a fatality rate of 2.38%, much higher than that on ordinary roads. Moreover, 45% of expressway traffic accidents are caused by weather factors, among which agglomerate fog are referred to as the ‘moving killer‘ on expressways. Essentially, agglomerate fog is a type of fog formed due to localized microclimate conditions, characterized by its small range (1−5 km), higher concentration, and difficulties in monitoring and prediction. Typically, visibility is clear outside the fog cluster, but inside, visibility drops significantly. The sudden change in visibility caused by fog clusters can easily lead to major traffic accidents, especially on highways. According to statistics from the Traffic Management Bureau of the Chinese Ministry of Public Security, the number of expressway sections prone to agglomerate fog increased from 1,468 in 2014–3,188 in 2018 (no data has been released since 2018) (https://www.gov.cn/xinwen/2018-11/14/content_5340443.htm), showing a year-by-year increasing trend. The hazards, monitoring and early warning, and causes of agglomerate fog have become research hotspots.

Many scholars have conducted research on the hazards and risks caused by agglomerate fog. For instance, Liu et al. employed the K-S algorithm and a multiple linear regression model to compare the impact of different visibility levels on vehicle speeds on expressways [1]. Tan et al. proposed a car-following model incorporating the risk illusion in foggy conditions, demonstrating that reduced visibility in foggy weather can lead to drivers misjudging the time-to-collision, thereby easily triggering traffic accidents [2]. Gao et al. analyzed the spatio-temporal characteristics, geographical environment, and weather background of agglomerate fog accidents using data from agglomerate fog-related traffic accidents and corresponding meteorological observations in Anhui Province. Their study provides a reference for agglomerate fog forecasting, early warning, and prevention [3]. Song et al. established a prediction model for the risk level of agglomerate fog-related accidents on expressways, based on accident information data caused by fog or agglomerate fog along expressways in Jiangsu and Anhui Provinces, China. This model contributes to providing meteorological support for traffic safety under adverse weather conditions [4]. Wang [5] and Niu [6], among others, explored the impact of agglomerate fog on expressway traffic safety by analyzing the characteristics of expressway sections prone to agglomerate fog, the frequency of agglomerate fog occurrence, and the number of accidents caused by agglomerate fog. Peng et al. analyzed the impact of fog-related visibility reduction on traffic parameters [7].

In order to mitigate the hazards of agglomerate fog various monitoring and early warning methods and facilities based on visibility meters, video imagery, the Internet of Things (IoT), and other technologies have been continuously developed. For instance, Li et al. proposed a agglomerate fog detection method based on a shallow convolutional neural network, which has been experimentally verified to achieve a detection accuracy of over 90% [8]. Geng et al. introduced a video image distance detection algorithm based on rectangular patterns, simplifying the traditional method of image calibration [9]. Yao et al. designed an early warning system for highway agglomerate fog monitoring robots based on BeiDou short messages and the MQTT protocol, enhancing the accuracy of agglomerate fog monitoring [10]. Peng et al. proposed a hybrid control framework that effectively mitigates shockwave propagation, alleviates traffic oscillations, and enhances road capacity [11].

Analysis of the causes of agglomerate fog serves as the foundation for research aimed at reducing its hazards and improving the accuracy of monitoring and early warning systems. Scholars have conducted studies on the causes of fog and agglomerate fog from various perspectives, including physics, meteorology, and influencing factors. For example, Gultepe et al. summarized achievements in the formation, development, and dissipation of fog, and discussed in detail the advancements in observational analysis, forecasting models, and remote sensing methods [12]. Weston et al. analyzed the impact of meteorological conditions on fog formation by exploring the microphysical characteristics of fog [13]. Mazoyer et al. through microphysical observations of fog, indicated that the size of aerosols is a primary factor influencing fog concentration [14]. Wang et al. found that longwave radiation, humidity, and wind all contribute to fog formation [15]. Liang et al., by analyzing the impact of temperature, relative humidity, and wind speed on agglomerate fog formation, demonstrated that large diurnal temperature variations, low-lying terrain, and poor air circulation are the causes of agglomerate fog [16].

Currently, research on the spatial heterogeneity of factors influencing agglomerate fog and the scales of their influence has not been thoroughly explored. Based on an analysis of the spatiotemporal distribution characteristics of agglomerate-fog -prone road sections, this paper intends to explore the impacts and scales of influence factors such as terrain and DEM, regional DEM differences, water system density, NDVI, regional LST, and regional precipitation on agglomerate fog occurrence from a spatial perspective using the GWR model and MGWR model, which are suitable for spatial characteristic analysis [17–19]. The objective is to discuss the spatial heterogeneity of influencing factors, thereby improving the accuracy of agglomerate fog monitoring and prediction and ensuring traffic safety.

## Research area and data

### Research area

**Fig 1:**
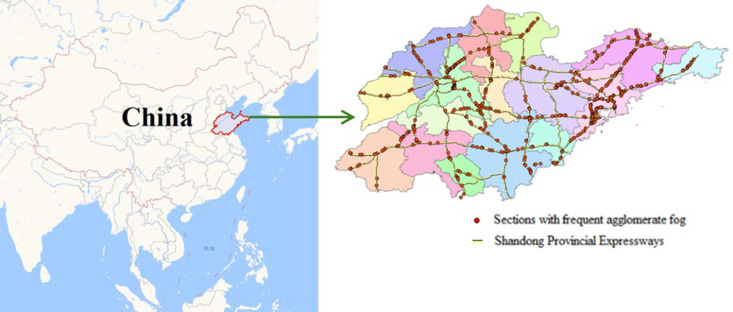
Research area. it sourced from Baidu Maps (https://map.baidu.com/). Shandong Province, located in the eastern coastal region of China at the lower reaches of the Yellow River (China’s second longest river), frequently experiences agglomerate fog on its expressways. The right figure shows the expressway (yellow line) and agglomerate fog-prone areas (red dots) with ≥3 annual occurrences (Derived from the Ministry of Public Security of China, https://www.gov.cn/xinwen/2018-11/14/content_5340443.htm).

This paper takes Shandong Province as the research area, as shown in Fig 1. Shandong Province is located along the eastern coast of China, in the lower reaches of the Yellow River (the second longest river in China), and has a warm temperate monsoon climate. Shandong Province governs 16 prefecture-level cities, covering an area of 158,100 square kilometers. By the end of 2023, its permanent resident population was 101.23 million. Shandong Province boasts developed transportation, with a highway network totaling 8,755 kilometers. The central part of Shandong Province is dominated by mountainous terrain, while the southwest and northwest are low-lying and flat, and the east features gentle hills with frequent occurrences of agglomerate fog. According to data derived from the Ministry of Public Security of China (https://www.gov.cn/xinwen/2018-11/14/content_5340443.htm), there are over 500 road segments with more than three occurrences of agglomerate fog annually.

### Data

#### Sections with frequent agglomerate fog in Shandong Province expressway.

The data on road segments with frequent agglomerate fog occurrences on Shandong expressways is sourced from the Traffic Management Bureau of the Ministry of Public Security of China (https://www.gov.cn/xinwen/2018-11/14/content_5340412.htm). Specifically, it includes the names of expressways with frequent agglomerate fog occurrences, the starting and ending points of these road segments, the months and time periods with frequent agglomerate fog, and the average annual number of occurrence days, as detailed in Table 1.

**Table 1:** Sections with frequent agglomerate fog.

Road section number	Expressway name	Expressway number	Starting point (km)	End point (km)	Frequent months	Frequent periods	Average number of occurrences per year (days)
1	Jinghu	G2	280	283	1-3, 11, 12	22:00 - 10:00	10
2	Jile	G2	301	353	11, 12,1, 2	7:00 - 9:00, 19:00 - 22:00	6
3	Jile y	G2	353	366	11, 12,1, 2y	7:00 - 9:00, 19:00 - 22:00	7
4	Jinghu	G2	400	402	3-5, 10-12	0:00 - 8:00	3
5	Jinghu	G2	404	406	3-5, 10-12	0:00 - 8:00	4

#### Geographic environmental data.

**Fig 2:**
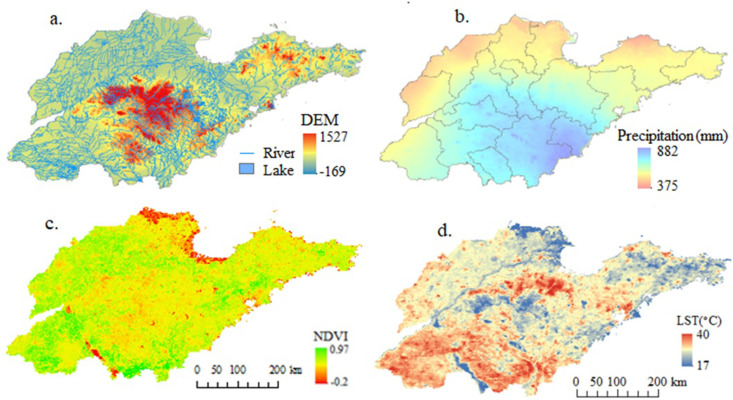
Geographical environment data of Shandong Province. **(A)** Digital Elevation Model (DEM) at 30-m resolution overlain with hydrological networks. The color gradient represents elevation variations, with warmer tones (red) indicating higher altitudes and blue depicting water systems. Data were obtained from the Resource and Environment Science and Data Center (RESDC; https://www.resdc.cn/). **(B)** Spatial patterns of mean annual precipitation, derived from the National Meteorological Information Center (NMIC; http://www.nmic.cn/). **(C)** NDVI and **(D)** LST (NDVI and LST data acquired from NASA’s official website: https://ladsweb.modaps.eosdis.nasa.gov/search/).

The geographical environment data utilized in this paper include: terrain data (digital elevation model (DEM), which represents ground elevation in the form of an ordered array of numerical values and reflects the topography, slope, and other information of the study area), water system, NDVI, where a higher value indicates better vegetation cover), andLST data, as shown in Fig 2.

#### Data preprocessing.

To analyze the relationship between geographical environmental factors such as terrain, water systems, vegetation (NDVI), temperature (LST), precipitation, and the frequent occurrence of agglomerate fog, this paper conducts statistics on the mean DEM, DEM elevation difference, water system density, NDVI, LST difference (using the MYD11A1 daytime and nighttime products from the Aqua satellite of MODIS), and precipitation data within a 1 km radius of highway sections prone to agglomerate fog in Shandong Province. These data are then matched one-to-one. Taking the data in Table 1 as an example, the data processing results are presented in Table 2.

**Table 2:** The result of data preprocessing.

Road section number	Express-way name	Express-way number	Starting point (km)	End point (km)	Frequent months	Frequent periods	Average number of occurrences per moths	Water system density (m^2^/km^2^)	DEM difference (m)	DEM	NDVI	LST difference (°C)	Precipitation (mm)
1	Jinghu	G2	280	283	1-3, 11, 12	22:00-10:00	5	0.22	5.2	35.2	0.32	22.21	56.74
2	Jile	G2	301	353	11, 12,1, 2	7:00-:00, 19:00-2:00	3	112	2.7	26.1	0.31	21.23	73.21
3	Jile	G2	353	366	11, 12,1, 2	7:00-9:00, 19:0-2:00	3	0.18	1.9	15.2	0.25	18.12	67.42
4	Jinghu	G2	400	402	3-5, 10-12	0:00-8:00	1	0.05	2.3	10.8	0.31	17.65	68.37
5	Jinghu	G2	04	406	3-5, 10-12	0:00-8:00	2	0.10	4.1	14.5	0.23	22.34	67.68

## Research methods

### Density analysis

Density analysis is a commonly used spatial analysis tool primarily used to measure and visualize the distribution density of geographical features (such as points or lines) within a specific area, thereby revealing the spatial relationships and potential patterns among these features. Kernel density analysis is a type of density analysis whose core principle lies in estimating the density of points using a kernel function(https://desktop.arcgis.com/zh-cn/arcmap/latest/tools/spatial-analyst-toolbox/kernel-density.htm). The choice of the kernel function and the setting of the bandwidth have significant impacts on the analysis results. Compared with traditional density analysis, kernel density analysis offers greater flexibility and accuracy, making it widely applicable in various fields such as hotspot analysis, resource allocation, environmental monitoring, urban planning, ecology, epidemiology, and more. In this paper, the Gaussian kernel function was selected for kernel density analysis, as shown in Equation 1.


$$\begin{array}{c} f(x)=(1/nh)*\Sigma k\left((x-x)i/h\right) \end{array}$$ (1)

Where: f(x) is the estimated density function, n is the number of sample points, h is the bandwidth, controlling the degree of smoothing, and k() is the kernel function.

### GWR model

This paper primarily analyzes the influencing factors and the causes of frequent agglomerate fog occurrences in Shandong Province from a spatial perspective. Therefore, the Geographically Weighted Regression (GWR) model, which is suitable for spatial analysis, was selected. GWR model embeds the spatial locations of the study objects into the regression parameters, as shown in Equation 2. GWR model takes into account the spatial local effects of the objects and can be used to quantify their spatial heterogeneity and spatial relationships. It is a highly effective method for analyzing data patterns with spatial characteristics and is therefore widely used in related fields involving spatial data and spatial pattern analysis.


$$y_{i}=\beta_{0}\left(u_{i},v_{i}\right)+\sum_{k=1}^{p}\beta_{k}\left(u_{i},v_{i}\right)x_{ik}+\varepsilon_{i}\;i=1,2,3\cdot \cdot \cdot ,n$$ (2)

Where: y denotes the value of the dependent variable at sample point i, $\beta_{0}\left(\mu_{i},v_{i}\right)$ represents the coordinates of sample point, $\beta_{k}\left(u_{i},v_{i}\right)x_{ik}$ is the kth regression parameter at sample point i, and $\varepsilon_{i}$ is the error correction term.

The regression parameters β for each sample point i are estimated using the local weighted least squares method on a point-by-point basis, where the weights are a function of the distance between the geographic spatial location of the regression point and the geographic spatial locations of other sample points (study objects). The weight function is sensitive to the bandwidth (typically representing the range of influence). The most commonly used methods for selecting the optimal bandwidth are Cross Validation (CV) and the Akaike Information Criterion (AIC).

In this paper, GWR is used to measure the relationship between natural environmental factors such as DEM, water systems, NDVI, LST, precipitation and road segments with frequent agglomerate fog occurrences.

### 2.3 MGWR model

The Multi-scale Geographically Weighted Regression (MGWR) model achieves the measurement of applicable scales for different variables by allowing each variable to have a different bandwidth (in contrast to the classic GWR, where all variables share the same bandwidth). The specific bandwidth for each variable can serve as an indicator of the spatial scale at which each spatial process operates.


$$y=\sum_{j=1}^{k}\beta_{bwj}x_{ij}+\varepsilon$$ (3)

Where: bwj represents the bandwidth of the regression coefficient for the jth variable, and $\beta_{bwj}$ denotes the regression coefficient of the jth variable at sample i.

Each regression coefficient $\beta_{bwj}$ in MGWR is obtained based on local regression, and the bandwidth is specific to each variable, which is the biggest difference from the classic GWR where all variables share the same bandwidth. In this paper, MGWR is used to measure the scope of influence of natural environmental factors such as DEM, water systems, NDVI, LST and precipitation on agglomerate fog.

## Analysis of results

### Temporal and spatial distribution characteristics of agglomerate fog

**Fig 3:**
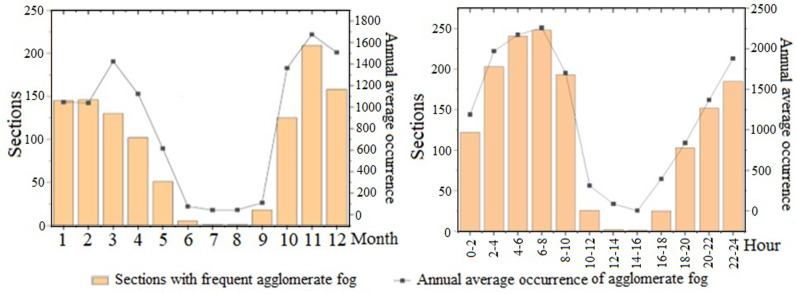
Time distribution statistics of agglomerate fog.

The segments of highways in Shandong Province prone to agglomerate fog were statistically analyzed by month and time period, as shown in Fig 3. Agglomerate fog occurred most frequently from October to April, with the highest incidence in November, affecting 237 road segments for a total of 1,966 occurrences. The number of agglomerate fog occurrences dropped sharply from May to September. In terms of time of day, agglomerate fog was most prevalent between 4:00 a.m. and 8:00 a.m., affecting 248 road segments with an average annual occurrence of 2,261 times. In summary, the occurrence of agglomerate fog exhibits strong regularity, primarily occurring during the early morning peak hours in autumn and winter when temperature differences are large.

**Fig 4:**
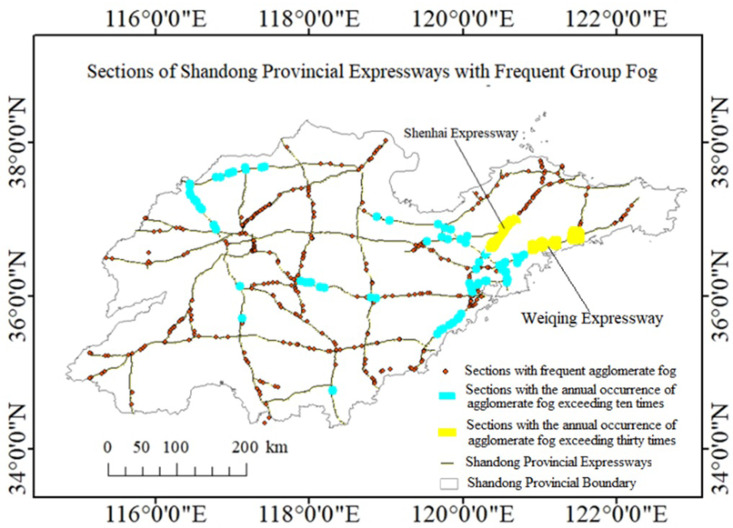
Statistical distribution of road segments prone to agglomerate fog.

There are over 500 road segments with an annual occurrence of more than 3 agglomerate fog events in Shandong Province, among which 125 road segments experience such events more than 10 times a year. The road segment with the highest frequency of agglomerate fog occurrence is the section from km 505 to km 550 of the Shenhai Expressway (Shenyang to Haikou), with an annual average of over 40 events. This is followed by the sections from km 100 to km 130 and from km 140 to km 170 of the Weiqing Expressway (Weihai to Qingdao), which experience an annual average of over 30 agglomerate fog events. This information is illustrated in Fig 4.

**Fig 5:**
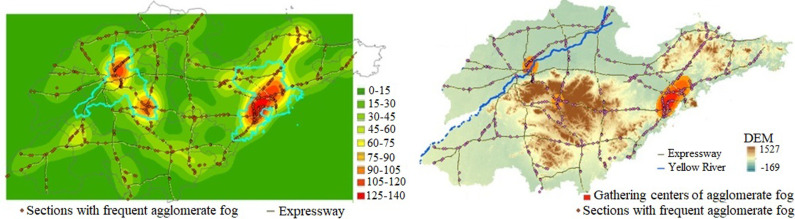
Results of Kernel density analysis.

Using Moran’s I index (a positive Moran’s I value indicates spatial positive correlation, while a negative value indicates spatial negative correlation) to conduct spatial autocorrelation analysis, we obtain a Moran’s I value of 0.86. This indicates that road segments prone to agglomerate fog exhibit strong spatial positive correlation and agglomeration. A kernel density analysis of these agglomerate fog-prone points in Shandong Province is shown in Fig 5a.

From Fig 5 above, it can be seen that there are three major high-density centers of agglomerate fog occurrences on highways in Shandong Province, located respectively in Qingdao City and Jinan City. The largest density center is formed around Jiaozhou Bay in Qingdao, involving highways such as the Shenhai Expressway, Weiqing Expressway, Jiaozhou Bay Expressway, and Qingyin Expressway. Another density center of agglomerate fog occurrences is formed near Donglv Expressway (Dongying to Lvliang) and the Jinan City Beltway Expressway in Jinan City. This center is surrounded by mountainous plains in central Shandong, including Mount Tai, Zulai Mountain, and Meng Mountain. The third density center is formed near the Jinghu Expressway, Qinglan Expressway, and Binlai Expressway in Jinan City. This center is located on the north side of Mount Tai, the highest mountain in Shandong, and the Yellow River, the largest river in Shandong, happens to pass through this density center.

From the above analysis, it can be seen that fog cluster-prone areas exhibit significant clustering, which provides important guidance for optimizing the layout of agglomerate fog monitoring equipment. For example, adding agglomerate fog monitoring, warning, and emergency guidance devices in high-frequency agglomerate fog areas can not only improve monitoring accuracy and reduce the probability of accidents but also minimize equipment investment.

### Analysis of influencing factors of Agglomerate fog based on GWR

To analyze the relationship between geographical environmental factors such as water systems, terrain, NDVI, temperature (LST), precipitation, and the frequency of agglomerate fog occurrences, this paper takes the data from November, which has the highest occurrence of agglomerate fog, as an example. Based on the GWR model, the occurrences of agglomerate fog on highways with high frequencies of agglomerate fog in Shandong Province are analyzed in relation to corresponding DEM, DEM difference, water system density, NDVI, LST difference, and precipitation. A comparison between the outputs of the six GWR models and the results of ordinary linear regression (OLS) is presented in Table 3.

**Table 3:** Comparison of GWR analysis results.

Variables	R^2^ based on GWR	Adjustd R^2^ based on GWR	R2 based on OLS	P
DEM	0.64	0.57	0.27	0.001
DEM difference	0.72	0.67	0.31	0.000
Water system density	0.48	0.41	0.19	0.001
NDVI	0.50	0.36	0.12	0.001
LST difference	0.68	0.61	0.19	0.001
Precipitation	0.45	0.39	0.21	0.001

As can be seen from Table 3, the accuracy of the analysis results based on the GWR model is much higher than that of the OLS results, due to the fact that the GWR model takes into account the local spatial effects of the sample points (Sections with frequent agglomerate fog occurrences). The frequency of agglomerate fog occurrences has a significant correlation with factors such as regional DEM, DEM difference, water system density, and LST difference, with the order of correlation being DEM difference > LST difference > DEM > NDVI > water system length > precipitation.

**Fig 6:**
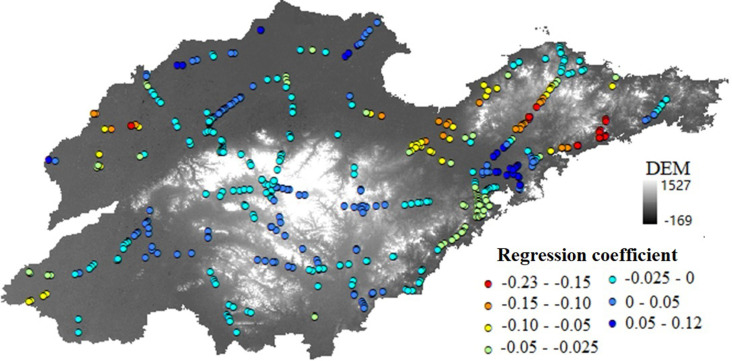
DEM regression coefficients based on the GWR model.

The OLS model assigns the same regression coefficient to all samples (in this case, the province), whereas the advantage of the GWR model lies in its ability to provide a separate regression coefficient for each sample point. This allows for the measurement of spatial variation in the influence of factors such as DEM, water system, and NDVI on the frequency of agglomerate fog occurrences. Taking the DEM difference as an example, see Fig 6.

From Fig 6, it is evident that in the analysis results of agglomerate fog occurrences and DEM based on the GWR model, each road segment prone to agglomerate fog has its own unique regression coefficient. A positive regression coefficient indicates a positive correlation between agglomerate fog occurrences and DEM on that road segment, suggesting that elevation promotes the occurrence of agglomerate fog. Conversely, a negative regression coefficient indicates a negative correlation, implying that DEM inhibits agglomerate fog occurrences in that area. In Fig 6, the elevation at the locations represented by dark blue sample points promotes agglomerate fog occurrences, while the other colored sample points indicate an inhibitory effect. Among them, 68.4% of the samples have negative regression coefficients, indicating that, overall, DEM has a predominantly negative correlation with agglomerate fog occurrences. However, for DEM difference, the opposite is true; 62% of the grid regression parameters in the analysis results of regional elevation difference are positive, showing a significant positive correlation. This means that as the elevation difference within a region increases, the frequency of agglomerate fog occurrences also rises.

In the analysis results of diurnal temperature variation (LST), more than 78% of the regression parameters are positive, suggesting that larger diurnal temperature variations promote the occurrence of agglomerate fog. For water system density, NDVI, and precipitation, more than 55% of the regression parameters are negative, indicating that they primarily have a negative impact. Overall, the negative effect regions of water systems on agglomerate fog occurrences are concentrated in the inland areas of Shandong Province, while the positive effect regions are in the coastal areas of Shandong Province. This may be because water systems have a role in regulating diurnal temperature variations, thus inhibiting the occurrence of agglomerate fog to a certain extent. Vegetation has a regulatory effect on both temperature and humidity, and NDVI also inhibits the occurrence of agglomerate fog to some degree. The negative effect regions of precipitation on agglomerate fog occurrences are concentrated in southern Shandong Province, while the positive effect regions are in the northern inland areas of Shandong Province. Single precipitation events have a greater impact on the formation of agglomerate fog, but this paper mainly analyzes from a spatial perspective. Instantaneous precipitation is difficult to spatialize, so the average precipitation in the region is selected instead.

### Analysis of influence scales based on the MGWR model

The MGWR model is utilized to measure the scope of influence of natural environmental factors such as DEM, water systems, NDVI, and LST on agglomerate fog occurrences. The MGWR model achieves the measurement of applicable scales for different variables by allowing each variable to have a different bandwidth (unlike the classic GWR where all variables share the same bandwidth). The specific bandwidth for each variable can serve as an indicator of the spatial scale of the effect of each spatial variable. The results are presented in Table 4.

**Table 4:** Results based on the MGWR model.

Variables	Influence scale(m)	R^2^ based on MGWR	Adjustd R^2^ based on MGWR	P
DEM	308	0.689	0.612	0.001
DEM difference	314	0.771	0.659	0.000
NDVI	44	0.637	0.595	0.001
Water system density	599	0.593	0.566	0.001
LST difference	108	0.710	0. 672	0.001
Precipitation	673	0.532	0.47	0.001

As shown in Table 4, the influence range (adaptive scale) of precipitation is the largest, extending to 673 meters, followed by water systems with an influence range of 599 meters, and then by DEM differences, which have an influence range of 314 meters. The smallest influence range is observed for NDVI, spanning only 44 meters, with LST differences coming in second smallest at 108 meters. The larger influence ranges of precipitation and water systems suggest weaker spatial heterogeneity in their impact on the occurrence of agglomerate fog. The limited influence range of NDVI, at just 44 meters, indicates strong spatial heterogeneity in vegetation cover along highways in Shandong Province. Additionally, Table 4 demonstrates that the fitting results of MGWR, after incorporating influence ranges, are superior to those of GWR, and it allows for individual examination of the spatial heterogeneity of different influencing factors.

Based on the analysis of influencing factors and their scales, it is possible to attempt to influence the occurrence of agglomerate fog in high-frequency areas by altering the surface environment. For instance, areas with large LST differences promote agglomerate fog formation, while NDVI inhibits agglomerate fog occurrence. Therefore, in agglomerate fog-prone areas, increasing vegetation coverage can effectively reduce LST differences and suppress the occurrence of agglomerate fog. According to the influence ranges of NDVI and LST (44 meters and 108 meters, respectively), the scope of vegetation greening on both sides of the highway can be rationally planned.

## Conclusion

To enhance the monitoring accuracy of agglomerate fog on expressways, this paper takes the frequently occurring agglomerate fog data on Shandong expressways as an example. Based on the analysis of the spatiotemporal distribution characteristics of agglomerate fog, from a spatial perspective, it employs GWR and MGWR models to analyze the influence and scale of factors such as DEM, DEM difference, water system density, NDVI, LST difference, and precipitation on agglomerate fog. The main research conclusions are as follows: Agglomerate fog frequently occurs in the early morning during autumn and winter when the temperature difference is large. Three concentration centers of agglomerate fog-prone road segments in Shandong expressways are identified, located near Jiaozhou Bay, within intermountain basins of the central region, and across the northern plain of Mount Tai (where the Yellow River traverses the concentration center). The impacts of various influencing factors on agglomerate fog are ranked as follows: DEM > DEM difference > LST difference > water system density > NDVI > precipitation, among which DEM difference and LST difference mainly promote the formation of agglomerate fog, whereas other factors generally exhibit inhibitory effect. The influence range (adaptive scale) of precipitation is the largest, at 673 meters, followed by the water system with an influence range of 599 meters, and NDVI shows the smallest influence range at only 44 meters. This study not only improves the accuracy and efficiency of agglomerate fog monitoring but also provides guidance for the layout of agglomerate fog monitoring and early warning equipment, as well as the greening along expressways. It holds significant importance for reducing the accident rate on expressways.
